# The gastric microbiome altered by A4GNT deficiency in mice

**DOI:** 10.3389/fmicb.2025.1541800

**Published:** 2025-02-12

**Authors:** Dawei Gong, Yuqiang Gao, Rui Shi, Xiaona Xu, Mengchao Yu, Shumin Zhang, Lili Wang, Quanjiang Dong

**Affiliations:** ^1^Dalian Medical University, Dalian, China; ^2^Department of Gastroenterology, The Fourth People’s Hospital of Jinan, Jinan, China; ^3^Central Laboratories, Department of Gastroenterology, Qingdao Municipal Hospital, Qingdao, China; ^4^Qingdao Medical College, Qingdao University, Qingdao, China

**Keywords:** *A4gnt*, gastric microbiome, *Desulfovibrio*, *Prevotellamassilia*, gastric cancer

## Abstract

**Background:**

Selective antimicrobial effects have been found for α1,4-linked N-acetylglucosamine residues at the terminus of O-glycans attached to a core protein of gastric gland mucin. *A4gnt* encodes α1,4-N-acetylglucosaminyl transferase, which is responsible for the biosynthesis of α1,4-linked N-acetylglucosamine. The impact of A4GNT on the establishment and homeostasis of the gastric microbiome remains to be clarified. The aim of this study was to characterize the gastric microbiome in mice deficient for the production of α1,4-linked N-acetylglucosamine.

**Methods:**

The gastric microbiome within *A4gnt*^−/−^ mice and wild-type mice was analyzed using high-throughput sequencing of bacterial 16S rRNA.

**Results:**

In *A4gnt*^−/−^ mice, which spontaneously develop gastric cancer, the community structure of the gastric microbiome was altered. The relative abundance of mutagenic *Desulfovibrio* and proinflammatory *Prevotellamassilia* in these mice was significantly increased, especially 4 weeks after birth. The co-occurrence network appeared to be much more complex. Functional prediction demonstrated considerable decreases in the relative frequencies of functions associated with polysaccharide metabolism and transportation.

**Conclusion:**

The distinct profile in *A4gnt*^−/−^ mice demonstrated a vital role of A4GNT in the establishment of the gastric microbiome. A dysbiotic gastric microbiome may contribute to the spontaneous development of gastric cancer in mice.

## 1 Introduction

The gut microbiome plays a vital role in human health and diseases (Nardone et al., 2017; Coker et al., 2018). Dysbiosis of the gut microbiome is involved in a variety of gut disorders and extra-gastrointestinal diseases (Umbrello and Esposito, 2016; Sgambato et al., 2017). In the human stomach, the biodiversity of the microbiome is altered in the context of gastric cancer (Aviles-Jimenez et al., 2014; Wang et al., 2020). Oral bacteria in the gastric microbiome appear to play an important role in the development of cancer (Coker et al., 2018). The structure of the microbial community seems to be disrupted in cancer (Wang et al., 2020). The involvement of the dysbiotic microbiome in the occurrence of gastric cancer has been considered, although definite carcinogenic bacteria remain to be identified. Development of the gut microbiome begins at birth and gradually yields a mature microbiome (Dominguez-Bello et al., 2010; D'Argenio and Salvatore, 2015; Tanaka and Nakayama, 2017). Human genetic background considerably affects the establishment and homeostasis of the gut microbiome. Deficiencies in the products of host genes alter the biodiversity and composition of the microbiome, ultimately leading to the development of cancer (Bali et al., 2021).

The gastric mucosa is covered by surface mucins and gland mucins (Hidaka et al., 2001). These mucins are key to maintaining the normal physiology of the stomach. Gastric gland mucin (MUC6) contains O-linked glycans attached to the core protein with terminal *α*1,4-linked N-acetylglucosamine (α-GlcNAc) residues (Zhang et al., 2001; Nordman et al., 2002). *A4gnt* encodes *α*1,4-N-acetylglucosaminyl transferase, which is responsible for the biosynthesis of α-GlcNAc. Selective antimicrobial effects have been found for *α*-GlcNAc at the terminus of O-glycans (Kawakubo et al., 2004). This effect was abolished by A4GNT deficiency. Terminal α-GlcNAc suppresses the growth of *Helicobacter pylori* and *Clostridium difficile* by inhibiting the biosynthesis of the bacterial cell wall constituent cholesteryl-α-D-glucopyranoside (Kawakubo et al., 2004). No antibacterial effect has been found for bacteria lacking cholesteryl-*α*-D-glucopyranoside, including *Escherichia coli, Pseudomonas aeruginosa, Klebsiella pneumoniae, Staphylococcus aureus, α-Streptococcus*, and *Streptococcus pneumoniae* (Kawakubo et al., 2004). The *A4gnt* gene is specifically expressed on the gastric mucosa and pancreatic duct surface (Kawakubo et al., 2004; Yamanoi and Nakayama, 2018). Reduced expression of this gene has been linked to an increased risk of precancerous diseases and gastric cancer (Ferreira et al., 2006). Deletion of *A4gnt* has been shown to induce a complete loss of α-GlcNAc expression and spontaneous development of gastric cancer, even in the absence of *H. pylori* infection in mice (Karasawa et al., 2012; Shiratsu et al., 2014). The gastric mucosa shows increased inflammation with elevated proinflammatory gene expression in mice deficient in A4GNT.

A few studies have been conducted to investigate the impact of host genetic background on the construction of the gastric microbiome. The cancer-associated *MUC1* variations are associated with an increased abundance of *Ochrobactrum* (Wang et al., 2020). In *MyD88* and *Trif*- double knockout mice, the profile of the gastric microbiome is altered (Bali et al., 2021). This might render these mice more susceptible to the development of gastric cancer upon *H. pylori* infection. Thus far, the impact of A4GNT on the establishment and homeostasis of the gastric microbiome remains unclear. In this study, we aimed to explore the profile of the gastric microbiome in the context of deficient production of *α*-GlcNAc, attempting to elucidate the impacts of A4GNT on the establishment and integrity of the gastric microbiome.

## 2 Materials and methods

### 2.1 Animals

All animal experiments were performed with the approval of the Laboratory Animal Care Committee of Qingdao University (2019-017A). The rearing of the mice was conducted according to the institutional rules following approval from the National Science and Technology Commission of China (2013 Second revision). Male wild-type (WT) C57BL/6 N mice were obtained from the Animal Central of Qingdao Municipal Hospital. *A4gnt*^−/−^ C57BL/6 N mice were constructed with CRISPR–Cas9 technology to target *A4gnt*, which was conducted by Cyagen Biosciences Limited Company. The genotypes of the mice were confirmed using multiplex PCR analyses of *A4gnt* alleles of tail DNA with allele-specific primers. Specifically, analysis using 3 primers (F1: 5′-TGGTTAGAATTGTTCGAGTAGGACT-3′, F2: 5′-ATAAAGAGGGCAAGACTGTGGTTA-3′, R1: 5′-CATGAGCATGGACTATAGACAGCA-3′) yielded a 520 bp amplicon for *A4gnt*^−/−^ mice, two amplicons of 520 bp and 458 bp for *A4gnt*^+/−^mice, and a 458 bp amplicon for WT mice. All mice were housed in autoclaved cages under specific pathogen–free conditions and supplemented with sterile food (Pengyue Laboratory Animal Breeding CO. Ltd., China) and water. Male *A4gnt*^−/−^ mice at 1 day, 3 days, 1 week, 4 weeks, and 10 weeks after birth (4 mice at each time point) were used in all experiments, and 20 age-matched WT mice were used as controls. After a 24-h fast, the mice were sacrificed by cervical dislocation. Gastric samples were collected under strictly sterile conditions and luminal contents of the stomach were washed off with sterile saline. Samples were stored at –80°C for genomic extraction of the gastric microbiome.

### 2.2 Analyses of the gastric microbiome

To analyze the microbial communities of the gastric mucosa in mice, genomic DNA was extracted from gastric mucosa samples as previously reported (Wang et al., 2016). The variable V3–V4 region of the 16S rRNA gene was PCR amplified with primers 338F/806R to generate the amplicon libraries. Sequencing was performed on a HiSeq 2,500 platform (Illumina, Hayward, CA, United States). A total of 4,052,236 paired-end reads were obtained. After quality control and filtration, 4,013,648 reads were produced with an average of 100,341 reads per sample. The reads were analyzed using UPARSE (Edgar, 2013). Following global trimming at 250 nucleotides, reads were dereplicated, and singletons were discarded. Subsequently, reads were clustered into operational taxonomic units (OTUs) assuming 97% identity. Chimeric reads were then removed. Rarefaction curves for the OTUs were constructed to estimate the sequencing depth for each individual sample (Supplementary Figure S1). Taxonomy assignment was performed using the Silva (Quast et al., 2013) and Greengenes databases (DeSantis et al., 2006) on QIIME2 platform.^1^ The annotation results were shown in Supplementary Table S1.

### 2.3 Statistical analyses

Analyses of alpha diversity of gastric microbiome in mice was conducted as described previously (Wang et al., 2016). The Mann–Whitney *U*-test was performed to detect significant differences in alpha diversity. *p*-values <0.05 were considered as statistically significant. The beta diversity of the gastric microbiome in mice was assessed using Principal Coordinates Analysis (PCoA) based on the weighted UniFrac distance. Comparisons of the relative abundances of taxa between groups were performed using version 1.0 of LEfSe (Linear discriminant analysis effect size) (Segata et al., 2011). An LDA value greater than 2 at a *p*-value <0.05 was considered statistically significant. To analyze the correlation network, Spearman correlations were computed between the genera in *A4gnt*^−/−^ or WT mice groups. The correlations that had absolute Spearman coefficient values greater than or equal to 0.6 with a *p*-value <0.05 were transformed into links between two genera in the genus network. Gephi software (0.9.2) was then used to construct network figures. To predict the functions of the microbial community, PICRUSt (v1.1.1) was used (Langille et al., 2013). The accuracy of the predicted metagenomes was assessed by the nearest sequenced taxon index (NSTI). Predicted functions were categorized with KEGG Orthology. STAMP (v2.1.3) was used to compare different groups and Benjamini-Hochberg correction method was used for multiple correction (Parks et al., 2014).

## 3 Results

### 3.1 Biodiversity and composition of the gastric microbiome

**Figure 1:**
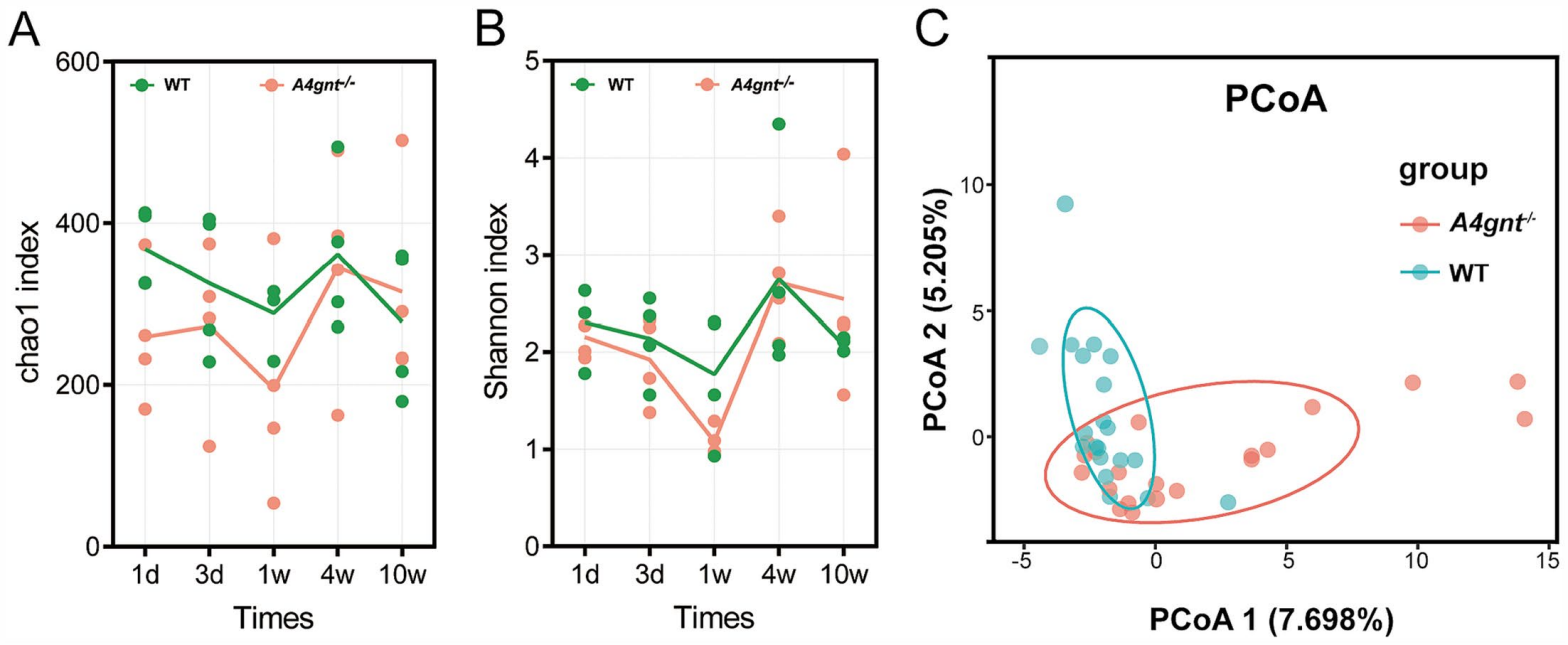
Biodiversity of the gastric microbiome in *A4gnt*^−/−^ and WT mice. The values of the Chao 1 index **(A)** and Shannon index **(B)** at different time points after birth in *A4gnt*^−/−^ and WT mice; dissimilarities in the structure of the gastric microbiome were assessed (*p* = 0.041) using PCoA **(C)**. WT, wild-type mice.

In *A4gnt^−/−^* mice, both the Chao1 and Shannon indices of the gastric microbiome were slightly lower than those in WT mice, but the difference was not statistically significant (Table 1). On the first day after birth, both the Chao 1 and Shannon indices were lower in *A4gnt*^−/−^ mice. A gradual increasing trend of the Chao1 and Shannon indices was found with increasing time in *A4gnt*^−/−^ mice, with the exception of a decrease at 1 week after birth (Figures 1A,B). In contrast, the trends of the Chao1 and Shannon indices were relatively stable in WT mice at different time points. PCoA of the gastric microbiome demonstrated that the community structure in *A4gnt*^−/−^ mice was distinct from that in WT mice (Figure 1C). These findings indicated that the establishment of the gastric microbiome in *A4gnt*^−/−^ mice was affected.

**Table 1:** Features of the gastric microbiome in *A4gnt^−/−^* mice.

Properties	*A4gnt^−/−^* mice (*n* = 20)	WT mice (*n* = 20)	*P*-values
Alpha diversity			
Chao1 index	283.85 ± 102.42	307.24 ± 88.32	>0.05
Shannon index	2.08 ± 0.79	2.20 ± 0.65	>0.05
Composition			
*Desulfovibrio*	0.05 ± 0.18%	0.002 ± 0.001%	0.027
*Prevotellamassilia*	0.12 ± 0.25%	0.01 ± 0.02%	0.035
*Staphylococcus*	0.02 ± 0.01%	0.12 ± 0.11%	0.038
*Turicibacter*	0.02 ± 0.01%	0.05 ± 0.03%	0.049
Co-occurrences			
Network number of nodes	26	25	–
Clustering coefficient	0.87 ± 0.13	0.33 ± 0.36	4.14E-09
Average degree	9.54 ± 5.09	2.56 ± 1.29	2.35E-08
Closeness centrality	0.76 ± 0.14	0.40 ± 0.25	6.80E-09
Predicted functions			
Polysaccharide metabolism	Decreased	Increased	–

–, not applicable; WT, wild-type mice.

**Figure 2:**
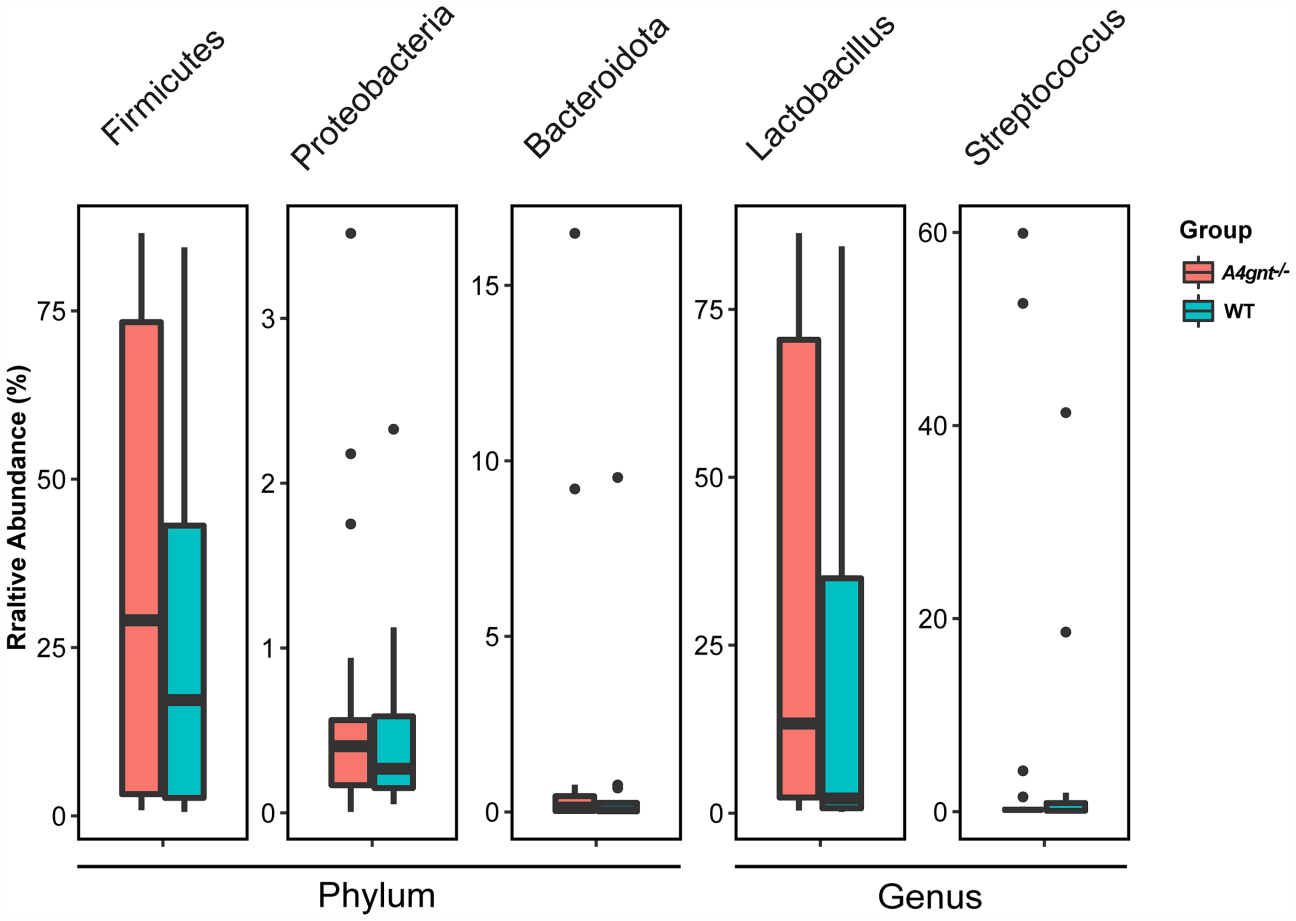
The relative abundance of domain dominant bacteria of the gastric microbiome in *A4gnt^−/−^* and WT mice at the phylum and genus level. The relative abundance of dominant phyla and genera showed no significant difference between *A4gnt^−/−^* and WT mice (all *p* values greater than 0.05) using Mann–Whitney *U test*. WT, wild-type mice.

**Figure 3:**
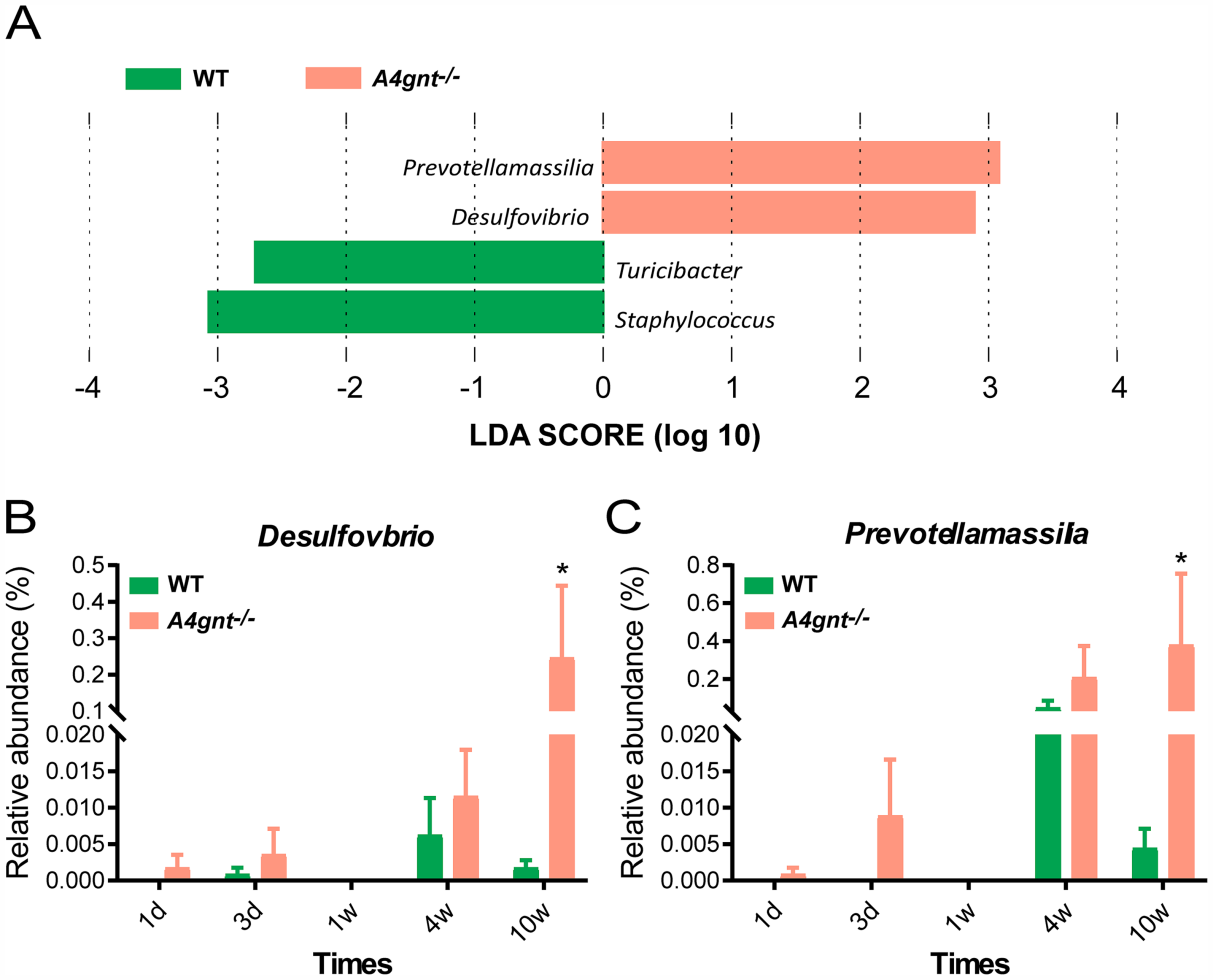
Analyses of the composition of the gastric microbiome. LEfSe analyses of the gastric microbiome in *A4gnt^−/−^* and WT mice by combining all time points. Bacterial genera enriched in *A4gnt*^−/−^ mice had a positive LDA score, while those enriched in WT mice had a negative score. Bacteria with LDA scores higher than 2 are shown **(A)**; variations in the relative abundance of *Desulfovibrio* **(B)** and *Prevotellamassilia* **(C)** across time points after birth in *A4gnt*^−/−^ and WT mice. * *p* < 0.05. WT, wild-type mice.

Compositional analyses showed a total of 35 phyla and 513 genera were detected in the gastric microbiom. The average number of bacterial phylum and genus per mice was 11 ± 4 and 71 ± 33, respectively. In both *A4gnt*^−/−^ and WT mice, analyses of bacterial relative abundance found Firmicutes, Bacteroidota and Proteobacteria were dominant phyla, while *Lactobacillus* and *Streptococcus* were dominant genera in the gastric microbiome (Figure 2; Supplementary Figure S2). These was no significant difference between *A4gnt*^−/−^ and WT mice in the relative abundance of these dominant phyla and genera. LEfSe analyses, which combined data from all time points, identified four genera with an LDA score greater than 2.0 between *A4gnt^−/−^* and WT mice (Figure 3A). The relative abundance of *Desulfovibrio* and *Prevotellamassilia* was increased in *A4gnt*^−/−^ mice, while *Staphylococcus* and *Turicibacter* were depleted (Table 1). The relative abundance of *Desulfovibrio* and *Prevotellamassilia* gradually increased in *A4gnt*^−/−^ mice from birth to 10 weeks. Furthermore, at 10 weeks after birth, the abundance of these bacteria was significantly higher in *A4gnt*^−/−^ mice compared to WT mice (*p* < 0.05 for both comparisons) (Figures 3B,C).

### 3.2 Co-occurrence network analyses

**Figure 4:**
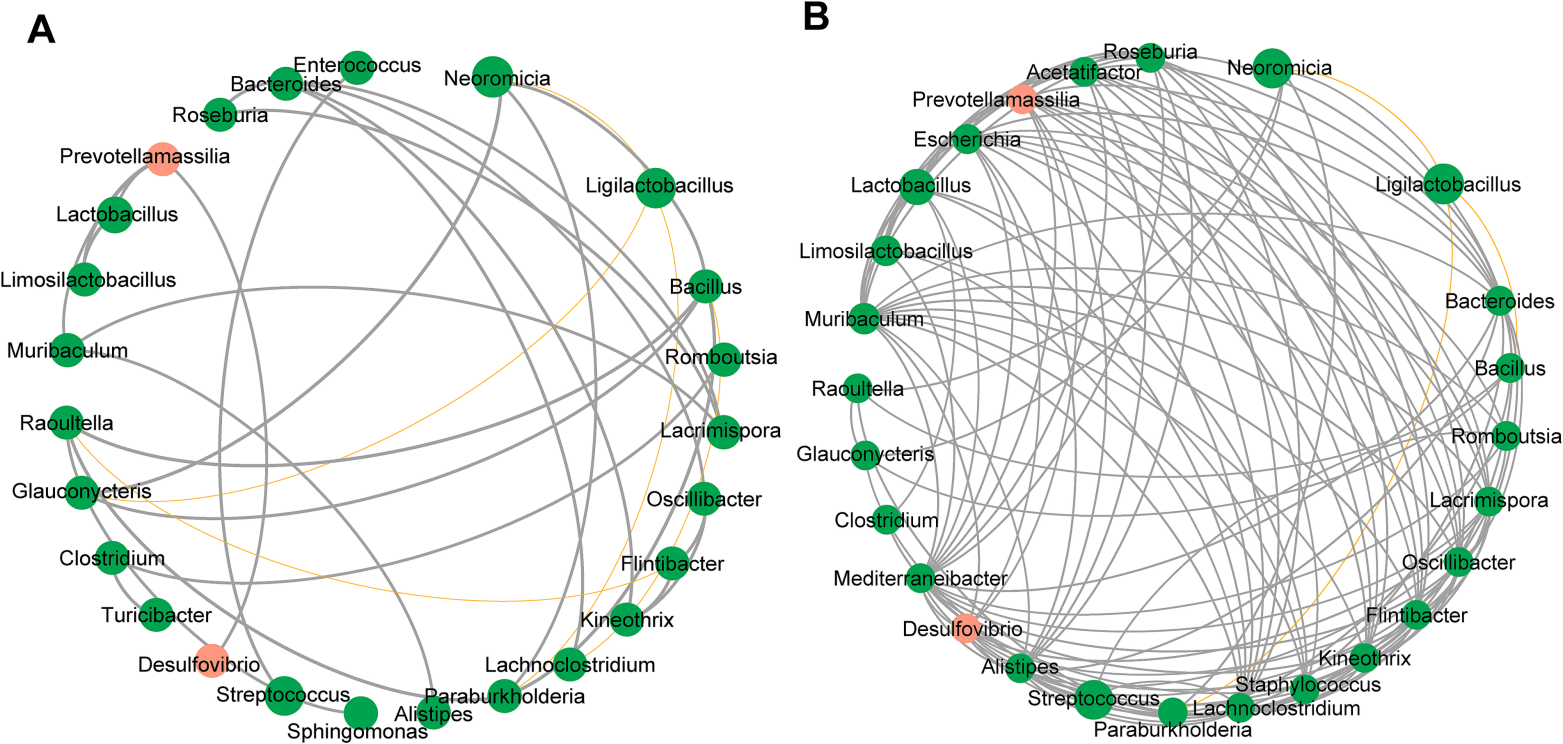
Correlation network of the gastric microbiome in WT **(A)** and *A4gnt*^−/−^ mice **(B)**. The correlation coefficient was calculated with Spearman’s rank correlation test (|r| ≥ 0.6). Gephi software (0.9.2) was used for network construction. Gray line: positive correlation; Yellow line: negative correlation.

The co-occurrence network of the gastric microbiome differed substantially between *A4gnt*^−/−^ and WT mice (Figures 4A,B). Although the number of nodes in the networks was similar in both groups of mice, the values of the clustering coefficient, average degree and closeness centrality were significantly higher in *A4gnt*^−/−^ mice (Table 1). This indicated much more complexity and much higher density of the co-occurrence network in *A4gnt*^−/−^ mice. Notably, the degrees of *Desulfovibrio* and *Prevotellamassilia* within the network of *A4gnt*^−/−^ mice were 4 and 9, respectively, markedly higher than those in WT mice (1 and 3, respectively). This indicates an increase in the interactions of both bacteria with other bacteria in the gastric microbiome.

### 3.3 Predicted functional capacities of the gastric microbiome

**Figure 5:**
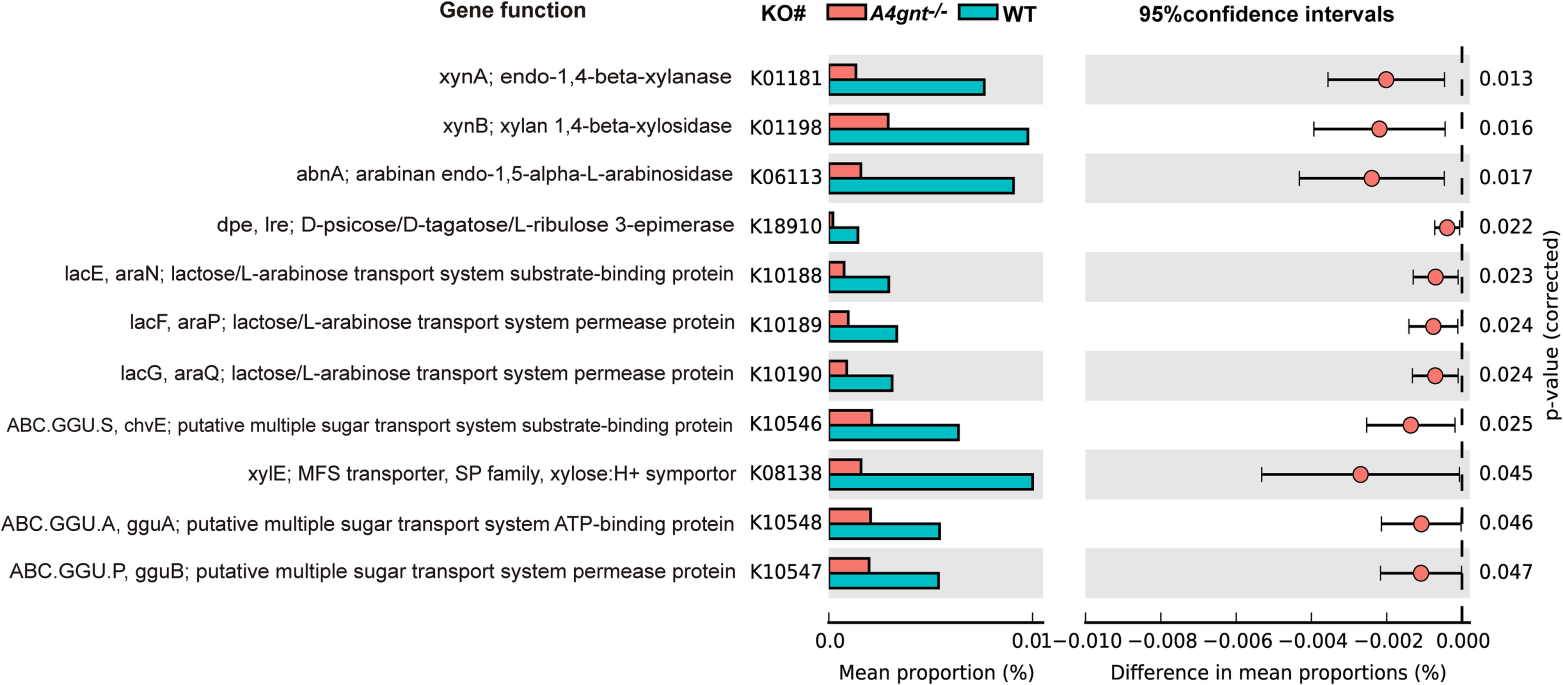
Functions of the gastric microbiome predicted using PICRUSt. Differences in predicted functions between groups were evaluated using STAMP. Statistical significance was considered to be indicated by *p* < 0.05. KEGG, Kyoto Encyclopedia of Genes and Genomes; WT, wild-type mice.

At levels 1, 2, and 3 of the KEGG pathway, the predicted functions of the gastric microbiome showed no significant difference between *A4gnt*^−/−^ and WT mice. However, functions related to carbohydrate metabolism and transport were substantially altered in *A4gnt*^−/−^ mice. The relative frequencies of functions associated with polysaccharide metabolism, including endo-1,4-beta-xylanase, xylan 1,4-beta-xylosidase, arabinan endo-1,5-alpha-L-arabinosidase and polygalacturonase, were significantly decreased in *A4gnt*^−/−^ mice. Similarly, the relative frequencies of the functions to transport lactose/L-arabinose, sugars and xylitol were also significantly decreased in the gastric microbiome of *A4gnt*^−/−^ mice (Figure 5; Table 1).

## 4 Discussion

It has been shown that *α*-GlcNAc at the terminus of O-glycans synthesized by A4GNT possesses great potential for inhibiting bacterial growth. However, it remains unclear whether A4GNT participates in maintaining the homeostasis of the gastric microbiome. Moreover, mechanisms of spontaneous development of gastric cancer in *A4gnt*^−/−^ mice require further exploration. In this study, we explored whether A4GNT deficiency in gastric mucosa cells would interfere with the establishment and structure of the gastric microbiome. Our results demonstrated that the structure of the microbial community in the stomach of *A4gnt*^−/−^ mice was distinct from that of WT mice. This indicates that deficiency in the activity of acetyl-glucosaminyl transferase considerably alters the normal structure of the gastric microbiome (Kawakubo et al., 2004). In *A4gnt*^−/−^ mice, both the Chao 1 and Shannon diversity indices tended to gradually increase from birth to the age of 10 weeks. This suggests that the loss of the capacity to suppress bacterial growth in *A4gnt*^−/−^ mice is likely to facilitate the growth of more bacteria, enhancing the biodiversity of the gastric microbiome (Zhang et al., 2001). Increased biodiversity of the gut microbiome has been found in *Muc2*^−/−^ mice (Wu et al., 2018). A higher biodiversity of the gut microbiome has been linked to the occurrence of colorectal cancer. Meanwhile, we found that the co-occurrence network of the gastric microbiome in *A4gnt*^−/−^ mice possessed much higher values for the clustering coefficient, average degree and closeness centrality, indicating more complex bacterial interactions in the microbial community. Although the alpha diversity of the gastric microbiome in *A4gnt*^−/−^ mice was slightly lower than that in WT mice, the difference was not statistically significant. It is suggested that complex bacterial interactions could partially explain the alteration in the structure of the gastric microbiome in *A4gnt*^−/−^ mice. Our results demonstrate that A4GNT deficiency appreciably impacts the gastric microbiome, most likely due to the effects of A4GNT on bacterial growth inhibition. This is consistent with findings from a study on antimicrobial peptide deficiency in mice that showed that the host ability to inhibit bacteria substantially impacts the gut microbial community (Yoshimura et al., 2018).

Compositional analyses of the gastric microbiome revealed that *Desulfovibrio* and *Prevotellamassilia* were enriched in *A4gnt*^−/−^ mice. While both genera remained at low levels in WT mice, their abundance sharply increased in *A4gnt*^−/−^ mice especially after 4 weeks. It was suggested that the two bacteria gradually played a significant role in the establishment of the gastric microbiome from birth onwards. The reasons for the increase in the abundance of these bacteria were unclear. One possible reason could be that these two bacteria are adapted to surviving in an A4GNT-deficient ecological environment. Increased abundance of *Desulfovibrio* has been observed in Muc2^−/−^ mice (Wu et al., 2018). The loss of bacteria-inhibiting activities and reduced amounts of carbohydrates available for bacterial growth may contribute to the enrichment of *Desulfovibrio*. In contrast, the depletion of *Staphylococcus* in *A4gnt*^−/−^ mice might be attributed to the reduced amount of *α*-GlcNAc accessible to the genus. *Staphylococcus* requires exogenous α-GlcNAc for construction of the cell wall (Kuroda et al., 2007). *Desulfovibrio* is a potential pathobiont. It has been shown that *Desulfovibrio* is enriched in the microbiome in the context of cancer and inflammatory diseases (Rowan et al., 2010; Thomas et al., 2016; Liu et al., 2021). A recent study reported that *Desulfovibrio* is especially enriched in stage IV gastric cancer, making it a candidate biomarker for predicting gastric cancer (Liu et al., 2021). An increased relative abundance of this bacterium is associated with colorectal cancer (Thomas et al., 2016; Deng et al., 2024). Furthermore, it is also enriched in the feces of patients with ulcerative colitis (Rowan et al., 2010). *Desulfovibrio* are sulfate-reducing bacteria and produce hydrogen sulfide (Singh and Lin, 2015). The bacterium causes genomic instability and DNA damage in epithelial cells, thus enhancing mutations in colon cancer (Muyzer and Stams, 2008; Zhu et al., 2014; Thomas et al., 2016). It promotes the production of Lipopolysaccharide, inducing acute inflammation and sepsis (Han et al., 2021). A novel mechanism by which *Desulfovibrio vulgaris*, a member of *Desulfovibrio*, contributes to the development of colorectal cancer has been studied. Through the interaction between its flagellin and leucine-rich repeat containing 19 (LRRC19), *Desulfovibrio vulgaris* activates the TNF receptor-associated factor 6 (TRAF6)/transforming growth factor-*β*-activated kinase 1 (TAK1) signaling pathway, thereby promoting epithelial-mesenchymal transition and driving the progression of colorectal cancer (Dong et al., 2025). *Prevotellamassilia* is a bacterium recently isolated from a melanoma patient (Ndongo et al., 2016). The bacterium is associated with immunotherapy-related colitis (Mao et al., 2021). Thus, the gastric microbiome in *A4gnt*^−/−^ mice appears to be enriched for the genera containing proinflammatory and mutagenic bacteria. Since the gastric microbiome is unstable from infancy to adulthood and its composition may vary greatly between individuals (Palmer et al., 2007), the potential contribution of these bacteria to gastric carcinogenesis remains to be validated in adult mice.

The results of PICRUSt analyses from this study revealed reductions in the relative frequencies of metabolic functions related to polysaccharides and cell transport capacity for monosaccharides or oligosaccharides. Endo-1,4-beta-xylanase, xylan 1,4-beta-xylosidase and arabinan endo-1,5-alpha-L-arabinosidase are produced by many gut bacteria to degrade environmental xylans into oligosaccharides and monosaccharides (Desai et al., 2016; Rohman et al., 2019; Pruss et al., 2021; La Rosa et al., 2022). They are subsequently transported into the bacterial periplasm and cytoplasm for the production of energy (Hespell and Whitehead, 1990; Sohail et al., 2022). The decrease in the relative frequencies of the degradation of xylan and transport of its potential products indicates a reduction in the utilization of xylan for energy production in the gastric microbiome of *A4gnt*^−/−^ mice. This probably reflects the compositional alterations caused by the absence of bacteria-inhibiting activities from *α*-GlcNAc attached to MUC6. In contrast, α-GlcNAc from glycans linked to mucins sustains the growth of gut commensals and maintains the homeostasis of the gut microbiome (Desai et al., 2016; Pruss et al., 2021). Many bacteria utilize exogenous *α*-GlcNAc as a nutrient or a constituent of the cell wall (Sa-Nogueira et al., 1997; Zeng et al., 2010; Bidart et al., 2018). In *A4gnt*^−/−^ mice, α-GlcNAc is absent from glycans linked to MUC6. A reduced amount of α-GlcNAc accessible to bacteria likely suppresses the growth of certain bacteria, thus disturbing the homeostasis of the gastric microbiome. A recent study reported that brown seaweed-derived *β*-glucan can potentially restrain the development of gastric dysplasia to mediate its tissue-preserving activity in *A4gnt ^−/−^* mice, which also supports our results (Desamero et al., 2018). *Desulfovibrio* is in mutualism with many bacterial species in the microbiome (Faith et al., 2011; Santana et al., 2012). Experimental colonization with *Desulfovibrio piger* in gnotobiotic mice led to alterations in the composition of an artificial human gut microbiome. Microbial RNA-Seq analysis showed that, in the presence of *D. piger*, genes encoding enzymes involved in carbohydrate metabolism, such as malate dehydrogenase, exhibited lower levels of expression compared to their absence (Rey et al., 2013). Therefore, an increased abundance of *Desulfovibrio* might contribute to the decreased frequency of the functions observed in our study by altering the composition and structure of the gastric microbiome in *A4gnt ^−/−^* mice. However, research on the metabolism of *Prevotellamassilia* is scarce, making it necessary to further explore its bacterial metabolic functions in order to understand their impact on the gastric microbiome.

## 5 Conclusion

In summary, our results demonstrated a distinct gastric microbiome in *A4gnt*^−/−^ mice. It was enriched in potential proinflammatory and genotoxic bacterial genera, resulting in enhanced pathogenic activities of the gastric microbiome. Loss of the bacterial suppressive activities and potential nutrient availability mediated by α-GlcNAc likely contribute to the reshaped bacterial interactions and network, leading to altered compositions of the gastric microbiome. The altered gastric microbiome may thus partially account for the development of gastric cancer in the context of deficiency of A4GNT. Further elucidation of the role and mechanisms of A4GNT in maintaining the homeostasis of the gastric microbiome is needed.

## Data Availability

The datasets presented in this study can be found in online repositories. The names of the repository/repositories and accession number(s) can be found below: NCBI/PRJNA875238.

## Footnotes

^1^ http://qiime.org/scripts/assign_taxonomy.html
