# Corrigendum: The gastric microbiome altered by A4GNT deficiency in mice

**DOI:** 10.3389/fmicb.2025.1604379

**Published:** 2025-04-22

**Authors:** Dawei Gong, Yuqiang Gao, Rui Shi, Xiaona Xu, Mengchao Yu, Shumin Zhang, Lili Wang, Quanjiang Dong

**Affiliations:** ^1^Dalian Medical University, Dalian, China; ^2^Department of Gastroenterology, The Fourth People's Hospital of Jinan, Jinan, China; ^3^Central Laboratories, Department of Gastroenterology, Qingdao Municipal Hospital, Qingdao, China; ^4^Qingdao Medical College, Qingdao University, Qingdao, China

**Keywords:** *A4gnt*, gastric microbiome, *Desulfovibrio*, *Prevotellamassilia*, gastric cancer

In the published article, there was an error in the order of affiliations 1 and 2. Instead of:

“^1^Department of Gastroenterology, The Forth People's Hospital of Jinan, Jinan, China

^2^Central Laboratories, The affiliated Qingdao Municipal Hospital of Dalian Medical University, Qingdao, China”, it should be:

“^1^Dalian Medical University, Dalian, China

^2^Department of Gastroenterology, The Fourth People's Hospital of Jinan, Jinan, China”.

The authors apologize for this error and state that this does not change the scientific conclusions of the article in any way. The original article has been updated.

