# Supplementary material for: The gastric microbiome altered by A4GNT deficiency in mice
Source: Front Microbiol. 2025 Feb 12;16:1541800. doi: 10.3389/fmicb.2025.1541800 (PMC11861098; doi:10.3389/fmicb.2025.1541800)
Supplement: Supplementary file 1 [file Data_Sheet_1.ZIP › Supplementary Figures.DOCX]

**Supplementary Figures**

**Supplemental Figure 1.** Rarefaction curves of each individual sample in both WT and *A4gnt*^–/–^mice.

**Supplemental Figure 2**. Hierarchically clustered heatmap analysis showing the percent abundance (log2 scale) at the phylum level **(A)** and for the top 50 genera **(B)** of the gastric microbiome in both WT and *A4gnt^–/–^* mice. Rows and columns are clustered using the Manhattan distance and average linkage.

**Supplemental Figure 1.**


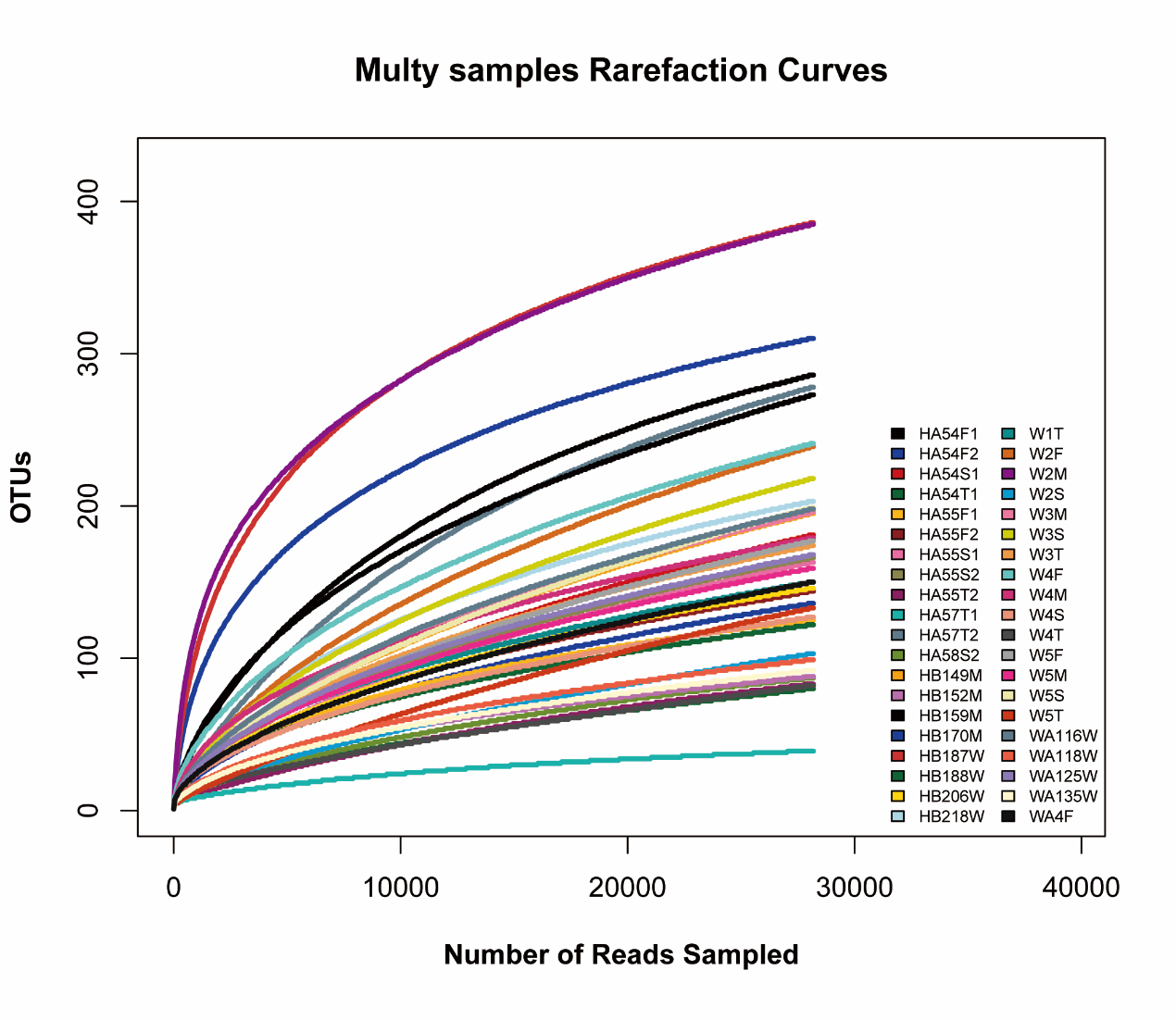


**Figure S1.** Rarefaction curves of each individual sample in both WT and *A4gnt*^–/–^mice.

**Supplemental Figure 2.**

**(A)**


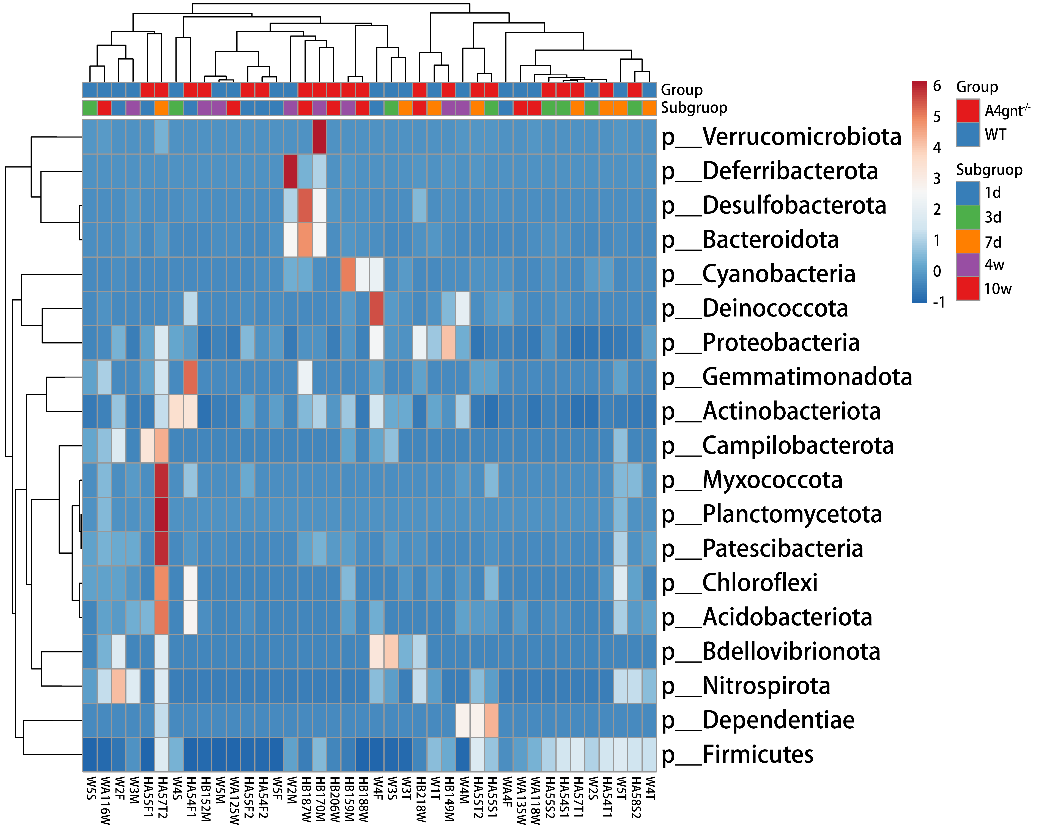


**(B)**

**
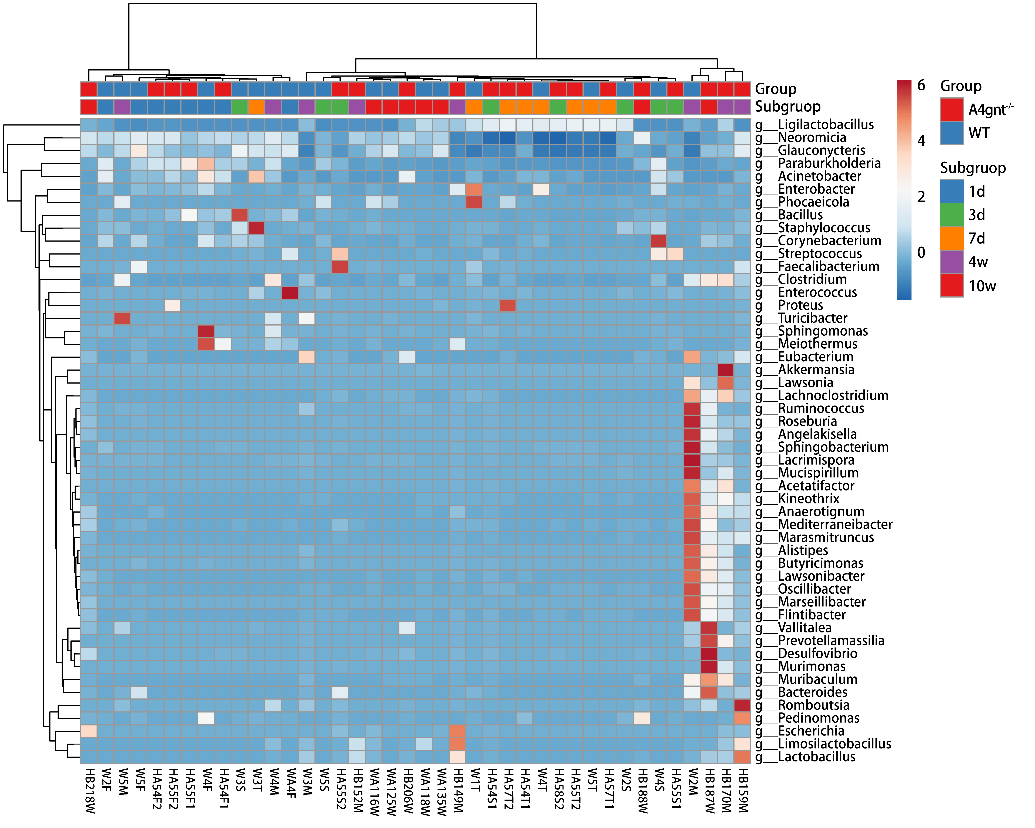
**

**Figure S2.** Hierarchically clustered heatmap analysis showing the percent abundance (log2 scale) at the phylum level **(A)** and for the top 50 genera **(B)** of the gastric microbiome in both WT and *A4gnt^–/–^* mice. Rows and columns are clustered using the Manhattan distance and average linkage.
